# Aggressive squamous cell carcinomas arising from mucosal lichen planus: A report of three cases

**DOI:** 10.1111/ddg.70174

**Published:** 2026-02-24

**Authors:** Neda Cramer, Dagmar Wilsmann‐Theis, Michael P. Schön, Jörg Wenzel, Julia Gallwas, Rotraut Mössner

**Affiliations:** ^1^ Department of Dermatology Venereology and Allergology University Medical Center Göttingen, Göttingen, Germany; ^2^ Center for Skin Diseases Department of Dermatology and Allergology University Hospital Bonn Bonn Germany; ^3^ Department of Gynecology and Obstetrics University Medical Center Göttingen Göttingen Germany

**Keywords:** Malignancy, Metastatic Squamous Cell Carcinoma, Mucosal Lichen Planus, Oral Lichen Planus, Vulvar Lichen Planus

Dear editor,

Lichen planus (LP) is a chronic inflammatory disease of the skin that can affect the mucous membranes, most commonly the oral or genital mucosa.^1^, ^2^ Mucosal LP has been discussed as a premalignant condition, and malignant transformation has been reported in 0.8% to 10% of patients with oral LP (OLP) and up to 2.5% with vulvar LP (VLP) in the literature.^3^, ^4^, ^5^ Thus, squamous cell carcinoma (SCC) arising from mucosal LP is a well‐known complication.

In this case series, we present three patients with metastatic SCC arising from mucosal LP, all of whom experienced a rapid disease course with a fatal outcome, intending to raise awareness of the potential for a fulminant, highly dynamic and metastatic course in mucosal LP (Table 1).

**TABLE 1:** Patient characteristics

	Patient 1	Patient 2	Patient 3
**Sex**	Female	Female	Female
**Age**	63 years	64 years	77 years
**Body mass index**	28.7	29.9	25.8
**Smoking status**	Never‐smoker	Never‐smoker	Never‐smoker, no alcohol
**Comorbidity**	Arterial hypertension, depression	Arterial hypertension, hypothyroidism, thrombocytopathy, depression	Type 2 diabetes mellitus, arterial hypertension, osteoporosis, colon carcinoma (2010)
**Age at onset of mucosal LP**	58 years	61 years	44 years
**Age at diagnosis of mucosal LP**	60 years	61 years	44 years
**Duration until diagnosis**	26 months	4 months	n. a.
**Affected mucous membranes**	Genital and oral	Genital	Oral and genital
**Extramucosal manifestation**	No	No	Yes (skin)
**Family history of mucosal LP**	Negative	Negative	Positive (daughter)
**Previous systemic therapies**	Intravenous dexamethasone pulse therapy Methotrexate Hydroxychloroquine	Acitretin	Oral prednisolone Acitretin
**Last therapy before diagnosis of malignancy**	Topical therapy	Acitretin	Topical therapy
**Interval between LP onset and first manifestation of malignancy**	2 years (dVIN)	2 years (vulvar carcinoma)	32 years (oropharyngeal carcinoma)
**Interval between diagnosis of malignancy and detection of metastases**	3 months	0 months	0 months
**Treatment after diagnosis of metastatic SCC**	Partial resection of the vulva and of the anterior vaginal wall, urethral resection, bilateral inguinal dissection Bilateral laparoscopic pelvic lymphadenectomy, bilateral adnexectomy Radiochemotherapy with cisplatin	Bilateral laparoscopic inguinal lymphadenectomy Chemotherapy with cisplatin‐paclitaxel Vulvectomy with bilateral inguinal lymphadenectomy Radiotherapy	Laser resection and bilateral neck dissection Left submandibular lymphadenectomy Radiochemotherapy with cisplatin
**Disease course**	2.5 months after completion of radiochemotherapy fulminant progression with cutaneous metastases, axillary lymph node metastases and peritoneal carcinomatosis	5 months after completing radiotherapy, extensive peritoneal carcinomatosis	13 months later presentation with inoperable, extensive local recurrence
**Deceased due to mucosal LP, age**	Yes, 63 years	Yes, 64 years	Yes, 77 years

*Abbr*.: dVIN, differentiated vulvar intraepithelial neoplasia; LP, lichen planus; n/a, not applicable; SCC, squamous cell carcinoma

Patient 1 is a 63‐year‐old woman with mucosal LP that manifested genitally 4.5 years ago, and orally five months later. Differentiated Vulvar Intraepithelial Neoplasia (dVIN) was first detected 1.5 years after disease onset and treated by her local gynecologist with laser vaporization. Then, immunosuppressive therapy for mucosal LP with intravenous dexamethasone pulse therapy and methotrexate was carried out for six months and was discontinued before a planned cholecystectomy. After 1.5 years, dVIN was detected again and treated. Three months later, a poorly differentiated non‐HPV‐associated SCC of the vulva was diagnosed. Staging examinations revealed suspicious inguinal and left pelvic lymph nodes and urethral infiltration. After surgery, radiochemotherapy with cisplatin was started. 2.5 months later, a fulminant progression with cutaneous metastases (lymphangiosis carcinomatosa, Figure 1a, b), peritoneal carcinomatosis and axillary lymph node metastases was observed. The patient deceased three weeks later.

**FIGURE 1:**
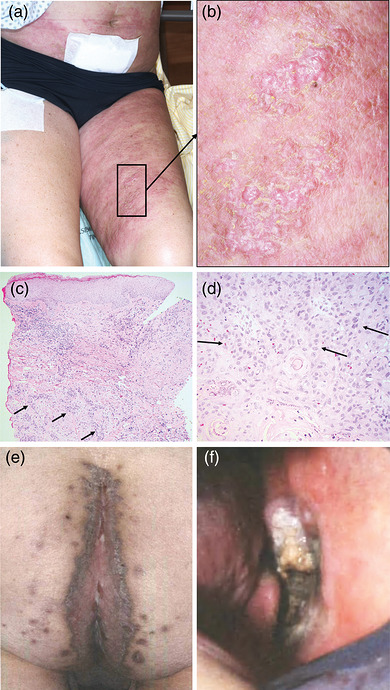
Clinical or histological images of patient 1, 2, and 3. Patient 1 with metastatic vulvar carcinoma, 2.5 months after surgery and radiochemotherapy with peritoneal carcinomatosis, axillary lymph node metastases and lymphangiosis carcinomatosa (a), the latter as a close‐up of the left ventromedial thigh (b). Histological images of patient 2: Underneath keratinized squamous epithelium without atypia is an infiltrating moderately differentiated squamous cell carcinoma (black arrows) that occupies the entire sample. The histological image shows the carcinoma at 5x (c), and 20x magnification (d). Patient 3 with pronounced anogenital lichen planus (e) at the time of diagnosis of oropharyngeal carcinoma on the left dorsal alveolar ridge (f).

Patient 2 is a 64‐year‐old woman who has had VLP for three years. Two years after diagnosis, under treatment with acitretin, a vulvar carcinoma arose from VLP (Figure 1c, d). A CT scan revealed a lymph node metastasis in the right groin and suspicious lymph nodes bilaterally in the parailiac region. She underwent bilateral laparoscopic inguinal lymphadenectomy followed by therapy with cisplatin‐paclitaxel, but her disease progressed. After vulvectomy with bilateral inguinal lymphadenectomy, the tumor bed and groin were irradiated. Approximately five months later, extensive peritoneal carcinomatosis occurred and she deceased.

Patient 3 is a 77‐year‐old woman with a history of LP for 33 years (Figure 1e). 32 years after diagnosis, oropharyngeal carcinoma arose from OLP (Figure 1f). Laser resection, neck dissection and left submandibular lymph node extirpation were performed, confirming moderately differentiated SCC and capsular perforation. Adjuvant radiochemotherapy with cisplatin was started but discontinued after four weeks due to severe nausea and diarrhea and grade III‐IV mucositis. 13 months later, histology and CT scan revealed an unresectable extensive local recurrence. Due to the previous radiation exposure, further radiotherapy was not performed. A palliative tracheostomy with a mucocutaneous anastomosis was carried out. The patient deceased 15 weeks later.

The potential for malignant development on the basis of an LP been increasingly addressed in case reports since the 1990s.^6^, ^7^, ^8^ Whether LP transforms directly into SCC, or chronic LP inflammation could drive carcinogenesis in a tumor that mutated independently of LP is still elusive and literature recognizes that both conditions are possible and may also coexist.^9^, ^10^, ^11^, ^12^, ^13^ Whole‐exome sequencing analyses indicate that cases of OLP that progress to oral SCC exhibit recurrent mutations in genes such as TP53, CELSR1, CASP8, and KMT2D, whereas these alterations are not detected in non‐transforming OLP lesions.^14^ Since molecular testing for these mutations is not yet clinically translated and validated for early detection, surveillance and histopathology remain the standard of care.^15^

Chronic inflammatory lesions of the vulva, such as mucosal LP and lichen sclerosus, may develop dVIN, which has a greater potential for progression to invasive SCC compared to other, HPV‐associated vulvar precancerous lesions, and also progresses more rapidly within three to twelve months.^16^, ^17^ Surgical excision is recommended as the treatment of choice for dVIN in the German and European guidelines.^18^, ^19^

In a prospective study involving 114 patients with VLP, one patient developed subsequent vulvar SCC.^20^ A cohort study found an increased risk of vulvar cancer in VLP patients.^21^ The largest case series of 38 LP‐associated vulvar SCCs showed a high rate of inguinal metastases at presentation (42%), recurrent vulvar carcinoma in the diseased mucosa (39%) and disease‐related death (37%).^22^ Such a fulminant and aggressive course was also observed in our two patients, who developed invasive vulvar carcinoma despite a short disease duration and with metastases only three months after diagnosis of dVIN in patient 1, and with metastases present already at diagnosis of vulvar carcinoma in patient 2. It should be noted that laser vaporization does not involve a histological examination, so it is not possible to retrospectively determine whether patient 1's vulvar carcinoma existed earlier. As opposed to vulvar carcinoma arising from mucosal LP, it has been suggested that oropharyngeal carcinoma arising from OLP lesions might have a less aggressive course compared to oropharyngeal carcinoma arising in non‐OLP patients, but data is sparse.^23^

Although there have been reports in the literature of patients with mucosal LP and resulting SCC, we would like to raise awareness of the potential for a fulminant, highly dynamic and metastatic course with a fatal outcome, especially in VLP. We would therefore like to emphasize that mucosal LP patients should receive interdisciplinary care and regular dermatological and gynecological check‐ups. This includes dermatological and gynecological examinations in which the skin, oral mucosa, genital region including the vulva and vagina, anal region, nails, and hair should be systematically assessed, and therapy‐resistant lesions should be biopsied regularly to rule out malignant transformation. Regarding specific examination intervals, the strongest evidence supports OLP every 3–4 months.^15^ The intervals for gynecological check‐ups are not uniformly specified in the literature. In our opinion, a check‐up every 3–4 months would also be advisable here, considering the fulminant course in the event of malignancy. Effective therapy of mucosal LP is needed to prevent potentially fatal malignant transformation of the lesions.

## CONFLICT OF INTEREST STATEMENT

J. Gallwas has been an advisor and/or received speakers’ honoraria or participated in clinical trials of the companies: MSD SHARP & DOHME, and Novartis Pharma.

R. Mössner has been an advisor and/or received speakers’ honoraria and/or received grants and/or participated in clinical trials of the following companies: Abbvie, Almirall, Biogen IDEC, Böhringer‐Ingelheim, Celgene, Janssen‐Cilag, Leo Pharma, Lilly, Moonlake, MSD SHARP & DOHME, Novartis Pharma, Pfizer, and UCB.

M. P. Schön has been an advisor and/or received speakers’ honoraria and/or received grants and/or participated in clinical trials of the following companies: AbbVie, Allmirall, Biogen, Böhringer‐Ingelheim, BMS, Celltrion, Janssen‐Cilag, Leo, Lilly, Novartis, Scinai, UCB.

J. Wenzel has received financial support (clinical studies, travel grants, speaker's fees) from GlaxoSmithKline, Novartis, Medac, Merck/Serono, Lilly, Bristol Myers Squibb, Roche, Actelion, Pfizer, Spirig, ArrayBio, Biogen, and Incyte.

D. Wilsmann‐Theis has been an advisor and/or received speakers’ honoraria or travel expense reimbursements and/or received grants and/or participated in clinical trials of the companies: AbbVie, Almirall‐Hermal, Amgen, Biogen, Bristol Myers Squibb, Boehringer Ingelheim,

Celgene, Forward Pharma, GlaxoSmithKline, Incyte, Janssen‐Cilag, Klinge Pharma, Leo, Eli Lilly, Medac, Merck, Moonlake, Novartis, Pfizer, and UCB.

N. Cramer reports no conflicts of interest.
